# The potential of biogas production and effects of alfalfa silage under the synergistic influence of *Lactobacillus acidophilus* and *Rosa roxburghii* pomace waste on the fermentation quality and bacterial community

**DOI:** 10.1128/msphere.01054-24

**Published:** 2025-05-06

**Authors:** Maoya Li, Jiachuhan Wang, Qiming Cheng, Zhongfu Long, Chao Chen, Yixiao Xie, Yao Lei, Yulian Chen, Yuanyuan Zhao, Xiangjiang He, Wei Yan, Zhijun Wang

**Affiliations:** 1 College of Animal Science, Guizhou University 655623, Guiyang, Guizhou, China; 2 Guizhou Institute of Prataculture, Guiyang, China; 3 Inner Mongolia Academy of Science and Technology, Hohhot, China; 4 College of Grassland, Resources and Environment, Key Laboratory of Grassland Resources of the Ministry of Education P.R. of China, and Key Laboratory of Forage Cultivation, Processing and High Efficient Utilization of the Ministry of Agriculture, Inner Mongolia Agricultural University 117454 , Hohhot, Inner Mongolia, China; Third Institute of Oceanography, Xiamen, China

**Keywords:** agricultural byproduct, lactic acid bacteria, *Rosa roxburghii *pomace, alfalfa, bacterial community, biogas production

## Abstract

The process of ensiling serves as a dual-purpose technique, functioning as a means of preserving biomass for biogas production while serving as a biological pretreatment method with the potential for little fermentation loss. In this study, we investigate the potential of *Rosa roxburghii* pomace (RP) and *Lactobacillus acidophilus* to improve the biomass preservation of biogas produced from alfalfa during anaerobic storage. Coinoculation of RP and *Lactobacillus acidophilus* resulted in better preservation of nutrients (biomass), with a significant increase of 47.38% within the lactic acid content and a notable decline of 40.34% in the ammonia nitrogen content relative to those in the control treatment. Moreover, coinoculation of RP and *Lactobacillus acidophilus* resulted in an elevated proportion of *Lactobacillus,* and the species *Lactiplantibacillus plantarum* dominated anaerobic fermentation. The synergistic effect of RP and *Lactobacillus acidophilus* continuously stimulated anaerobic fermentation, leading to 33.33% and 23.17% increases in methane production and acetate content, respectively, after 72 h of coinoculation with RP and *Lactobacillus acidophilus* compared to the control treatment. Overall, coinoculation of RP and *Lactobacillus acidophilus* offers an attractive opportunity to increase methane production from fruit waste while reducing costs and synergistically integrating with other pretreatment techniques to optimize the methane generation potential.

**IMPORTANCE:**

Considering the increasing global energy demand and urgent environmental issues, exploring prospective resources for bioenergy production is imperative. However, the biomass of legume perennials may serve as an inexpensive and stable source of clean energy for modern society due to its wide availability and broad range of sources. In addition, the combination of RP and *Lactobacillus acidophilus* application increased the abundance of Lactobacillus, inhibited the growth of Kosakonia, and promoted anaerobic fermentation, which had beneficial synergistic effects on biomass retention and biogas production in alfalfa samples. Coinoculation improvements with RP and *Lactobacillus acidophilus* observed here are expected to reduce costs associated with CH_4_ conversion bioprocesses and increase CH_4_ production.

## INTRODUCTION

The utilization of biomass has gained importance as a viable and renewable substitute for energy, hence offering potential contributions to the creation of sustainable biogas through anaerobic digestion (1). The importance of sustainable biogas production in ongoing efforts to combat worldwide warming and climate variability is noteworthy. This is due to its role as an alternative to fossil fuels, its ability to reduce the energy demands of waste treatment facilities, and its potential to generate valuable organic fertilizers (2). Considering the increasing global energy demand and urgent environmental issues, it is imperative to explore prospective resources for bioenergy production. Consequently, it is essential to promote sustainable bioenergy production from biomass. Worldwide, a wide range of agricultural materials have been utilized to generate methane. The enhancement of bioenergy production by anaerobic digestion can be achieved by implementing codigestion practices involving agricultural biomass wastes with high carbon content (3). For example, the inoculation of *Lactiplantibacillus paraplantarum* during the anaerobic preservation of corn results in an increase in acetic acid (AA) concentrations, hence facilitating the generation of biogas (4). As stated by Liu et al. (5), applying food waste to giant reeds promotes an anaerobic digestion process which ends up in an augmentation of biogas production. Boonpiyo et al. (6) reported that the co-digestion of Napier silage and food residue is an efficient method to enhance methane production. Larsen et al. (7) identified the potential of co-ensiling straw and sugar beet leaves in the form of biological pretreatment to promote methane production from straw. However, the biomass of legume perennials may serve as an inexpensive and stable source of clean energy for modern society due to its wide availability and broad range of sources (8).

Alfalfa (*Medicago sativa L*.) contains a substantial protein content and other elements that make it excellent for serving as a feeding product for livestock (9). The properties of alfalfa not only make it ideal for animal fodder but also make it a superior feedstock for biogas production (10–12). Alfalfa, with its significant nutritional content, may increase methane production when employed as a substrate in biogas generation, rendering it to emerge as a valuable resource in the realm of biogas production (8). These findings demonstrate that alfalfa possesses considerable biomethane potential and can be utilized as a viable feedstock in the production of biogas (8). One of the primary obstacles encountered in the production of biogas from alfalfa is an insufficient amount of water-soluble carbohydrates (WSC), which may result in limited generation of volatile fatty acids (VFAs) during the ensiling process. However, fruit pomace added for coensiling with alfalfa cannot only provide a portion of the WSC but also provides a significant amount of dietary fiber on its own. A multitude of investigations have provided evidence that the utilization of fruit waste may have value-added commodities, such as biofuels, fertilizers, and livestock feed (2, 13, 14). Previous investigations have indicated that optimal methane production can be attained by anaerobically storing a combination of citrus waste and corn in a 1:1 ratio (1). *Rosa roxburghii*, a plant rich in vitamin C, flavonoids, superoxide dismutase, and amino acids, is grown in Guizhou Province, China, which accounts for 80% of the cultivation area of *Rosa roxburghii* (14 × 10^4^ hm^2^ in 2022). *Rosa roxburghii* possesses a diverse array of bioactive constituents, comprising minerals, polysaccharides, phenolic compounds, triterpenes, and organic acids. These chemicals contribute to their remarkable functional efficacy, surpassing that of alternative fruits (15, 16). With increasing recognition for its functional activity, the *Rosa roxburghii* processing industry is also growing, but the processing of *Rosa roxburghii* also leads to the production of fruit residue as a byproduct. Moreover, *Rosa roxburghii* pomace (RP) is not easily preserved and is perishable, leading to resource waste and environmental pollution, and addressing this problem has emerged as a critical issue. Therefore, considering the principle of circular economies and the fact that the WSC present in RP can compensate for the lack of WSC in alfalfa, RP can be mixed with alfalfa for biogas production, thereby maximizing the reuse of this product. Nevertheless, there is a lack of comprehensive research on the biogas potential of RP. The process of ensiling serves as a dual-purpose technique, functioning as a means of preserving biomass for biogas production while serving as a biological pretreatment method with the potential for little fermentation loss (4, 5, 7). Ensiling, an anaerobic process, involves the partial degradation of structural polysaccharides (such as lignin, hemicellulose, and cellulose) present in plant biomass. The process of degradation is facilitated by lactic acid bacteria (LAB), which can metabolize WSC and generate intermediate substrates that are subsequently utilized in the methanogenesis phase, including lactic acid (LA) and VFAs (9). Then, the VFAs are converted to biogas. Consequently, biogas production can be improved. LAB are extensively exploited in silage production, and to guarantee good fermentation quality, more intermediate substrates used in the methanogenic step are produced (9). *Lactobacillus acidophilus* was found to be a biogas-producing bacterium. The process of biogas production is concomitant with the generation of other valuable metabolites, specifically organic acids, such as pyruvic, propionic (PA), AA, butyric (BA), formic acids, and LA (17). Investigations have established that *Lactobacillus acidophilus* can increase gas production from corn stover and rice stover (18).

Consequently, this research aimed to assess the bioaugmentation impacts of RP and LAB alone or in association with the bacterial community composition and the synthesis of VFAs in alfalfa when stored anaerobically for biogas generation.

## MATERIALS AND METHODS

### Microbial preparation

*Lactobacillus acidophilus* was supplied by Guangzhou Huawei Biotechnology Co., Ltd. Before ensiling, the bacteria were cultivated and enumerated on MRS medium (catalog number 027315, obtained from Aiyan Biological Co., Ltd., Shanghai, China). Subsequently, they were diluted with distilled water to the appropriate concentration. The strain dosage used for each treatment in the ensiling process of this study was 10 mL/kg fresh weight (FW).

### Processing of alfalfa samples

Alfalfa was harvested on 11 July 2022, in Anshun City, Guizhou Province. RP was obtained from Guizhou Hengliyuan Natural Biological Technology Co., Ltd., in Guiyang City, Guizhou Province, China. Table 1 illustrates the chemical components of alfalfa and RP. Vacuum packing equipment (14886, Zhejiang Deli Group Co. Ltd.) was used to seal 400 g of uniformly mixed chopped alfalfa into 2 cm × 30 cm polyethylene bags. The experimental conditions for the alfalfa samples were as detailed below: (i) CK (without additives), applied as 5% FW distilled water; (ii) R, applied as 5% FW RP (Guizhou Hengliyuan Natural Biological Technology Co., Ltd., Guizhou, China); (iii) L, applied as *Lactobacillus acidophilus* at 2 × 10^7^ cfu/g FW (Guangzhou Hua Microbial Technology Co., Ltd., Guangzhou, China); and (iv) RL, combined with the application of 5% FW *Rosa roxburghii* pomace and *Lactobacillus acidophilus* at 2 × 10^7^ cfu/g FW (Guangzhou Hua Microbial Technology Co., Ltd., Guangzhou, China). Three identical polyethylene bag samples of treatment samples were stored in the dark at room temperature (22°C–25°C). From each treatment, three polyethylene bags were selected for analysis of their chemical composition and fermentation quality at various time intervals: 1, 3, 7, 15, and 50 days. In addition, the bacterial community composition was assessed at 3, 15, and 50 days. Furthermore, after a period of 50 days, the bags were examined for *in vitro* digestibility, gas production, and methane emissions.

**TABLE 1:** Chemical compositions of fresh alfalfa and *Rosa roxburghii* pomace^*a*^

Item	Fresh alfalfa	*Rosa roxburghii* pomace
Dry matter (% FM)	27.88 ± 1.32	29.55 ± 1.67
Crude protein (% DM)	26.64 ± 0.57	6.21 ± 0.43
Neutral detergent fiber (% DM)	43.51 ± 0.00	46.65 ± 0.03
Acid detergent fiber (% DM)	29.21 ± 0.08	25.14 ± 0.06
Water-soluble carbohydrate (% DM)	4.62 ± 0.09	5.03 ± 0.04

^*a*^ Data are expressed as the mean ± standard error. DM, dry matter; CP, crude protein; NDF, neutral detergent fiber; ADF, acid detergent fiber; WSC, water-soluble carbohydrate; FM, fresh matter.

### Chemical analyses of alfalfa samples

Each alfalfa sample was measured to obtain an amount of 20 g, which was then carefully mixed with 180 mL of distilled deionized water. The mixture was subsequently disrupted using a wall-breaking machine and filtered through four layers of coarse 7.5 × 7.5 cm cotton cloth (Hai Shi Hai Nuo Group Co., Ltd., Shandong, China). The filtrate that underwent filtration was utilized to quantify fermentation indicators such as organic acids present in the alfalfa samples. To determine the concentration of ammonia nitrogen (NH_3_-N), the sodium hypochlorite-phenol method was used, as described in the research carried out by Broderick et al. (19). The concentrations of LA, AA, PA, and BA were quantified using high-pressure liquid chromatography following the protocols outlined by Jia et al. (20). To ascertain the dry matter (DM) content, fresh alfalfa and alfalfa that had undergone anaerobic preservation were subjected to drying in an oven operated at a temperature of 65°C. Prior to analysis, the dried alfalfa samples were pulverized and filtered through a screen measuring 1 mm before being subjected to examination (Dahan Vibration Machinery Co., Ltd., Henan, China). This preparation was necessary to assess the levels of WSC, crude protein (CP), neutral detergent fiber (NDF), and acid detergent fiber (ADF) present within the samples. The method of anthrone, which was described by Owens et al. (21), was carried out to determine the concentration of the WSC. The CP content was examined with a Kjeldahl analyzer in accordance with the protocols established by the procedures of the Association of Official Analytical Chemists (22). This study used the methodology proposed by Van Soest et al. (23) to evaluate the levels of both NDF and ADF.

### Bacterial community analyses of alfalfa samples

The extraction of microbial DNA from the alfalfa samples (four treatments × three repetitions) was performed using the methodology outlined in Chen et al. (24). Genomic DNA was extracted from the samples utilizing the CTAB method and subsequently assessed for purity and concentration via agarose gel electrophoresis. An aliquot of the sample was transferred to a centrifuge tube and diluted to a concentration of 1 ng/µL with sterile water. Employing this diluted genomic DNA as a template, polymerase chain reaction (PCR) amplification was conducted using barcode-labeled specific primers, Phusion High-Fidelity PCR Master Mix with GC Buffer (New England Biolabs), and a high-efficiency, high-fidelity DNA polymerase. Following quantification of the PCR products, an equal amount of each was mixed thoroughly, and the resultant mixture was validated by 2% agarose gel electrophoresis. DNA ligase was then utilized to ligate sequencing adapters to the termini of the amplified DNA fragments. These fragments were purified and selected using AMpure PB magnetic beads to construct the SMRT Bell library. After resuspending the purified fragments in the buffer, size selection was performed using BluePipin, followed by a second purification step with AMpure PB magnetic beads. The library was quantified using a Qubit fluorometer, and the insert size was confirmed using an Agilent 2100 bioanalyzer. Sequencing was subsequently carried out on the PacBio platform. The resulting data, in bam format, were exported from the PacBio platform and demultiplexed based on barcode sequences using lima software. The sequences were then corrected with CCS (SMRT Link v7.0), filtered for SSRs, and primers were removed using cutadapt to yield the raw data. Through the use of certain primers, namely, 1514R and 27F, it was possible to accomplish amplification of the whole 16S ribosomal RNA (rRNA) gene. Each primer set incorporated Illumina barcode sequences to enable multiplexing of each sample. The sequencing of the amplicon library was performed using the Illumina MiSeq platform. The NCBI database already includes the raw sequencing data under accession number PRJNA1007581. The NovoMagic platform has been utilized to conduct canonical correlation analysis (CCA), principal coordinates analysis (PCoA), and additional analyses, along with visualizing the results through imaging. The calculation of observed OTUs, Shannon, and ACE index has been performed using QIIME software (version 1.9.1). The vegan package provides the bioenv function, which enables the identification of environmental components or combinations that exhibit the strongest correlation (Spearman) with the species matrix. Subsequently, a focused CCA could be conducted for the filtered environmental factors.

### Gas production characterization of *in vitro* rumen anaerobic fermentation of alfalfa samples

The *in vitro* anaerobic fermentation experiment went ahead complying with the approach presented by Menke et al. (25). Following the collection of rumen fluids from a sample of five bulls that were fitted with rumen fistulas, the fluids were then filtered through a triple layer of medical gauze. This was done before the morning feeding. A mixed rumen inoculum was produced by mixing the rumen filtrate and the artificial buffer solution (400 mL of distilled water, 0.1 mL of trace element solution, 200 mL of buffer solution, 200 mL of macroelement solution, 1 mL of resazurin solution, and 40 mL of reducing agent) in a ratio of 1:3 (vol/vol) and then flushing the mixture with carbon dioxide at a temperature of 39°C. This was done to keep the environment completely anaerobic throughout the preparation process. The alfalfa samples that were stored under anaerobic conditions were precisely weighed to 0.20 g. Afterward, the specimens were introduced into filtration bags alongside two glass beads. Subsequently, the bags were tightly sealed applying a sealer, and then transferred to a 150 mL glass syringe. The rumen inoculum was expeditiously moved to a glass syringe, which was then sealed and subjected to sequential incubation for 72 h set at 39°C. Each run involved the preparation of 36 sample bottles (four treatments × 3 replicates × 2 samples) and an additional three syringes as blank controls (containing only rumen inoculum). The scale readings of each syringe were recorded at regular intervals of 6, 12, 24, 36, 48, 60, and 72 h to determine the total gas output (mL) at each respective time point. Furthermore, as part of the investigation on the production of methane, a syringe was used to collect 5 mL of gas at each time point. There was also a collection of the fermentation broth at each time point, which was then utilized to determine the content of VFAs. The method employed in this study for assessing the *in vitro* digestibility of alfalfa samples was based on the approach published by Contreras et al. (26), with specific adaptations. After securing 1 g of the pulverized sample in each filter bag with heat, submerging it in acetone, desiccating it at 65°C for 24 h, and subsequently weighing it. The rumen inoculum (100 mL per bag) was added to each bag before being plunged into a preheated, CO_2_-filled serum container. As blank controls, three empty bags were inserted into the serum bottles. After 72 h of incubation at 39.0 ± 0.5°C, fermentation was stopped. Once the cleaned water becomes clear, extract the filter bag and cleanse it by rinsing it in hot distilled water in preparation for further analysis. Sun et al. (4) provided the data used to calculate methane production. According to the description of Li et al. (27), gas chromatography was used to measure the VFA concentrations. According to Meale et al. (28), the filter bags were heated at a temperature of 65°C for a period of 48 h to assess the ruminal DM digestibility of the alfalfa samples. The formula for the degradation rate of feed samples is as follows: DM digestibility = (*A*1 − *A*0)/*A*1 (DM digestibility: the degradation rate of the tested sample at a certain time point, in percent; *A*1: the DM mass of the substrate placed in the fermentation bottle, g; *A*0: the DM mass of the residue, g). Following that, the bags were placed in a desiccator for a minimum of 30 minutes to cool down, and then they were weighed.

### Statistical analysis

For each set of three replicate measurements, compute the mean and standard deviation, subsequently employing the analysis of variance (ANOVA) technique to rigorously assess all experimental data, with statistical significance determined at the *P* < 0.05 level. All graphical representations were generated using GraphPad Prism 9.0 to ensure professional data visualization.

## RESULTS AND DISCUSSION

### Chemical composition of alfalfa and *Rosa roxburghii* pomace prior to anaerobic storage

The chemical compositions of alfalfa and RP before anaerobic storage have been displayed in Table 1. Before anaerobic storage, the DM levels of alfalfa and RP were 27.88% and 29.55%, respectively, indicating that they could be used for LA fermentation. The NDF levels of alfalfa and RP were 43.51 and 46.65% DM, and the ADF levels were 29.21 and 25.14% DM, respectively (4). Our alfalfa material was similar to those of Huo et al. (29), which indicates that *in vitro* rumen anaerobic fermentation can be performed. It was determined that the WSC content of raw alfalfa was 4.62% DM, surpassing the WSC content of alfalfa utilized in the investigation presented by Zhang et al. (9). However, the research carried out by Cai et al. (30) provided evidence that freshly harvested forage containing a WSC proportion (>5% DM) ensures the quality of subsequent fermentation, thus requiring the addition of material containing higher WSC levels than alfalfa (4.62% DM). By contrast, the WSC content of RP was 5.03% DM, which provided a greater source of WSC for the anaerobic fermentation of alfalfa.

### Chemical composition of alfalfa samples during anaerobic storage

The term anaerobic digestion refers to a biological phenomenon wherein a variety of microorganisms decompose complex organic substances, including proteins, lipids, polymeric sugars, and sugar oligomers, under oxygen-deprived conditions. This process yields intermediate amounts of VFAs, while organic matter undergoes hydrolysis and fermentation, contributing to the creation of carbon dioxide, hydrogen, methane, and other byproducts (31). Good nutrient retention during fermentation can provide more substrate for biogas production (31). As anaerobic fermentation proceeded, the levels of DM, CP, NDF, ADF, and WSC (Table 2) in all the treatments progressively decreased due to the microorganisms utilizing the nutrients in the alfalfa samples to produce substances such as LA and VFAs (4). The L and RL treatments resulted in higher CP contents than the CK treatment did, indicating that they inhibited the degradation of proteins in alfalfa. Several factors contribute to the observed phenomenon. One possible explanation is the expeditious creation of a low-pH environment through the introduction of *Lactobacillus acidophilus*, which effectively suppresses protease activity and subsequently diminishes protein hydrolysis (24). In addition, the occurrence of tannins in RP might result in their binding to proteins as complexes, causing protein-polyphenol interactions that also hinder protein breakdown (1, 24). The outcomes of our investigation align with the preliminary results of Chen et al. (24), who observed that both *Lactiplantibacillus plantarum* treatment and coinoculation of *Lactiplantibacillus plantarum* and sea buckthorn pomace resulted in greater CP content than the CK treatment did. The findings of our study line up with the investigations undertaken by Sun et al. (4), who demonstrated that treatments that produced more methane also resulted in a higher CP content. The L and RL treatments resulted in lower NDF contents than the CK treatment did, which coincides with the outcomes of Zhang et al. (9). They discovered a decrease in NDF concentration in alfalfa silage when *Lactiplantibacillus plantarum* A1 and grape pomace were introduced as inoculants. Research revealed that the coensiling of wheat straw and sugar beet leaves resulted in a decrease in the NDF concentration, hence promoting the formation of biogas in the following processes (7). The RL-treated samples exhibited the lowest concentration of ADF, which aligns with the outcomes of Chen et al. (32), who published comparable results in alfalfa silage treatments coinoculated with gallnut tannins and *Lactiplantibacillus plantarum*. In addition, the elevated levels of NDF and ADF in R-treated samples could be linked to the substantial presence of lignin in the RP. There is a possibility that the higher WSC content that was discovered in the RP is the reason for the potential cause for the increased WSC content observed in the R and RL treatments. The findings validate that the combination of *Lactobacillus acidophilus* with RP treatment effectively enhances the preservation of nutrients in alfalfa samples.

**TABLE 2:** Chemical composition of alfalfa samples during 50 days of anaerobic storage^*a*^

Item	Treatment (T)	Ensiling period (D)					Mean^T^	SEM	*P* value		
		Day 1	Day 3	Day 7	Day 15	Day 50			T	D	T × D
DM, % FM	CK	27.67^Ac^	27.66^Ac^	26.44^Bc^	26.43^Bc^	26.26^Bc^	26.89				
	R	30.64^Aa^	30.21^ABa^	29.45^BCa^	28.73^Ca^	29.12^Ca^	29.63	0.058	<0.001	<0.001	0.002
	L	28.61^Ac^	28.51^ABb^	27.85^BCb^	27.61^Cb^	27.34^Cb^	27.97				
	RL	29.30^Ab^	29.25^Aa^	28.25^Ba^	28.14^Bab^	27.57^Bb^	28.46				
	Mean^D^	28.98	28.63	28.18	27.76	27.65					
CP, % DM	CK	23.73	22.71	22.15	22.04	21.97	22.52^c^				
	R	23.84	22.99	21.56	22.42	20.94	22.35^c^	0.063	<0.001	<0.001	0.101
	L	26.12	25.44	24.51	24.06	23.46	24.72^a^				
	RL	24.69	24.34	23.97	23.5	23.11	23.92^b^				
	Mean^D^	24.6^A^	23.87^B^	23.05^C^	23.00^C^	22.37^D^					
NDF, % DM	CK	44.67	44.38	42.66	41.7	40.67	42.82^a^				
	R	46.35	44.57	44.00	43.5	41.71	44.03^a^	0.276	0.001	<0.001	0.901
	L	43.37	42.00	41.33	39.73	38.37	40.96^b^				
	RL	42.67	42.38	40.47	40.03	38.39	40.79^b^				
	Mean^D^	44.27^A^	43.33^AB^	42.12^B^	41.24^B^	39.79^C^					
ADF, % DM	CK	30.91	30.14	30.67	30.67	29.00	30.28^a^				
	R	32.00	31.51	30.67	30.00	28.00	30.44^a^	0.252	0.01	<0.001	0.785
	L	31.33	30.47	28.67	27.33	27.23	29.01^ab^				
	RL	31.33	28.29	28.67	28.00	25.00	28.26^b^				
	Mean^D^	31.4^A^	30.1^AB^	29.67^B^	29^B^	27.31^C^					
WSC, % DM	CK	4.00	3.43	2.79	2.62	1.75	2.92^b^				
	R	4.99	4.44	3.65	3.04	2.54	3.73^a^	0.049	<0.001	<0.001	0.894
	L	4.6	3.57	2.96	2.38	1.81	3.06^b^				
	RL	5.03	4.01	3.36	2.84	2.18	3.48^a^				
	Mean^D^	4.66^A^	3.86^B^	3.19^C^	2.72^D^	2.07^E^					

^*a*^ A–E, means of ensiling days within a row with different superscripts differ in the same additive treatment (*P* < 0.05); a−d, means of additive treatments within a column with different superscripts differ in the same ensiling days (*P* < 0.05). CK, without additives; R, *Rosa roxburghii* pomace; L, *Lactobacillus acidophilus*; RL, combined *Rosa roxburghii* pomace with *Lactobacillus acidophilus*; DM, dry matter; CP, crude protein; NDF, neutral detergent fiber; ADF, acid detergent fiber; WSC, water-soluble carbohydrate; FM, fresh matter; SEM, standard error of the mean; T, treatment; D, ensiling days; T × D, interaction of treatments and ensiling days; SEM, standard error of the mean.

### Fermentation characteristics of alfalfa samples during anaerobic storage

The fermentation quality indicators of the alfalfa samples are presented in Table 3. The pH of the CK, L, and RL treatment samples decreased continuously during anaerobic storage, with the RL treatment sample exhibiting the fastest decrease in pH. The decrease in pH in alfalfa samples is a result of acid production during fermentation. The outcomes of our study align with those of Zhang et al. (9) for alfalfa. They observed that the mixed treatment with grape pomace and LAB had the lowest pH. Table 3 demonstrates that LA, AA, and PA dominate the anaerobic storage process. During anaerobic storage, the LA content of the CK, R, and RL treatment samples increased continuously. The LA content in the alfalfa samples was considerably enhanced (*P* < 0.05) in both the L and RL treatments compared to the CK treatment. In the L and RL treatments, the added *Lactobacillus acidophilus* is a homofermentative LAB, which can produce more LA under anaerobic conditions, thus resulting in a lower pH value (15). Notably, the RL treatment resulted in the highest observed LA content. The outcomes of our investigation are consistent with the findings presented by Chen et al. (24) for alfalfa. They reported that the mixed treatment of wet sea buckthorn pomace and LAB had the highest LA content. This finding suggested that L and RP synergistically affected LA yield during the anaerobic storage process. Furthermore, the bioactive compounds in PR may inhibit harmful bacteria and reduce competition between harmful bacteria and *Lactobacillus acidophilus*, thereby increasing the LA yield of alfalfa (15). The AA content increased during prolonged anaerobic storage. Previous findings have shown that inoculation with homofermentative LAB promotes homofermentation and reduces the AA content (33). Nevertheless, the AA content was notably higher in the alfalfa samples treated with R, L, and RL compared to those in the CK treatment group. This observation aligns with the outcomes of Chen et al. (24), who discovered that the incorporation of sea buckthorn pomace residue into alfalfa silage also increases the AA content. Research has demonstrated that the simultaneous digestion of large amounts of reed and food waste ends up in a notable augmentation in the AA content (5). An increase in the AA concentration has been demonstrated to significantly improve the generation of biogas in subsequent anaerobic digestion procedures (14). We hypothesize that the bacterial community analysis subsequently conducted on the alfalfa silage samples has revealed the presence of certain heterofermentative LAB, including *Lactobacillus buchneri* and *Lactobacillus brevis*, which are capable of converting LA to AA. The incorporation of *Lactobacillus acidophilus* and RP has led to an increased LA content, thus providing a more abundant substrate for the conversion to AA, ultimately resulting in a subsequent elevation of AA levels. Moreover, the PA content gradually increased during anaerobic storage, except in the R treatment. According to Jahanzad et al. (34), the AA and PA levels increased with increasing anaerobic storage. However, the absence of BA in every alfalfa sample indicates the success of the anaerobic storage process. Our findings contradict those of Sun et al. (4), who observed BA production, while no BA was detected in our study, suggesting that our study greatly limited clostridial fermentation, preventing BA production (35). This could be due to the pH decreasing so quickly that Clostridia cannot ferment sugar to BA or convert LA to BA (36). The concentration of NH_3_-N serves as a crucial biomarker for the degradation of chemical compounds known as CP. During anaerobic storage, the NH_3_-N content of all the alfalfa samples increased continuously as a result in part to the proliferation of unwanted bacteria such as *Bacillus* and *Clostridium*, as well as the enzymatic activity of plant proteolytic enzymes during fermentation, which continuously produce NH_3_-N (24). During anaerobic storage, the L and RL treatments contained lower levels of NH_3_-N compared to the CK treatment. Among the treatment samples, the RL treatment had the lowest NH_3_-N content. Our findings seem to correspond with the outcomes of Zhang et al. (9), which showed that the treatment with the maximum gas generation had the lowest NH_3_-N level. This suggests that RP and *Lactobacillus acidophilus* have a synergistic effect and that the bioactive compounds present in PR hinder the growth of undesirable bacteria, minimize the competition between harmful bacteria and *Lactobacillus acidophilus*, and promote the dominance of *Lactobacillus*, thereby impeding the development of bacteria responsible for CP degradation.

**TABLE 3:** Fermentation characteristics of alfalfa samples during 50 days of anaerobic storage^*a*^

Item	Treatment (T)	Ensiling period (D)					Mean^T^	SEM	*P* value		
		Day 1	Day 3	Day 7	Day 15	Day 50			T	D	T × D
pH	CK	5.98^Aa^	5.77^Ba^	5.64^Ca^	5.43^Da^	5.29^Ea^	5.62				
	R	5.68^Ab^	5.61^Ab^	5.27^Bb^	5.18^Bb^	5.19^Bb^	5.39	0.007	<0.001	<0.001	<0.001
	L	5.24^Ac^	5.10^Bc^	5.05^Cc^	4.98^Dc^	4.92^Ec^	5.06				
	RL	5.19^Ac^	5.08^Bc^	5.05^Bc^	4.90^Cc^	4.66^Dd^	4.98				
	Mean^D^	5.52	5.39	5.25	5.12	5.02					
LA, % DM	CK	1.32^Dc^	1.52^Db^	1.76^Cb^	2.03^Bc^	2.41^Ab^	1.81				
	R	1.52^Eb^	1.75^Db^	2.04^Cb^	2.16^Bc^	2.77^Ab^	2.05	0.029	<0.001	<0.001	0.032
	L	2.44^Ca^	2.60^Ca^	3.02^BCa^	3.38^ABb^	3.91^Aa^	3.07				
	RL	2.40^Ca^	2.85^Ca^	3.49^Ba^	3.75^Ba^	4.58^Aa^	3.41				
	Mean^D^	1.92	2.18	2.58	2.83	3.42					
AA, % DM	CK	0.78	0.79	0.88	0.91	0.98	0.85^c^				
	R	0.87	0.86	0.9	0.94	1.03	0.92^b^	0.007	<0.001	<0.001	0.085
	L	0.84	0.87	0.92	0.95	1.01	0.95^b^				
	RL	0.92	0.97	0.97	1.03	1.11	0.99^a^				
	Mean^D^	0.88^B^	0.87^B^	0.91^B^	0.97^A^	1.00^A^					
PA, % DM	CK	0.00	0.12^Cc^	0.26^Bbc^	0.45^Aab^	0.49^Ab^	0.26				
	R	0.00	0.41^a^	0.40^a^	0.49^ab^	0.51^b^	0.36	0.008	<0.001	<0.001	<0.001
	L	0.00	0.14^Cbc^	0.18^Cc^	0.37^Bb^	0.58^Ab^	0.25				
	RL	0.00	0.20^Db^	0.34^Cab^	0.57^Ba^	0.71^Aa^	0.37				
	Mean^D^	0.00	0.22	0.30	0.47	0.57					
NH_3_-N, % TN	CK	1.58^Cb^	3.74^Ba^	4.10^Ba^	7.23^Aa^	7.14^Aa^	4.76				
	R	1.73^Da^	2.54^Cb^	4.33^Ba^	4.49^Bb^	6.41^Aab^	3.9	0.062	<0.001	<0.001	<0.001
	L	1.32^Ec^	2.25^Dbc^	3.26^Cb^	4.42^Bb^	5.87^Ab^	3.42				
	RL	1.34^Ec^	2.06^Dc^	2.50^Cc^	3.17^Bb^	4.26^Ac^	2.67				
	Mean^D^	1.49	2.74	3.46	4.83	5.92					

^*a*^ A–E, means of ensiling days within a row with different superscripts differ in the same additive treatment (*P* < 0.05); a−d, means of additive treatments within a column with different superscripts differ in the same ensiling days (*P* < 0.05). CK, without additives; R, *Rosa roxburghii* pomace; L, *Lactobacillus acidophilus*; RL, combined *Rosa roxburghii* pomace with *Lactobacillus acidophilus*; DM, dry matter; LA, lactic acid; AA, acetic acid; PA, propionic acid; BA, butyric acid; NH_3_-N, ammonia nitrogen; FM, fresh matter; SEM, standard error of the mean; T, treatment; D, ensiling days; T × D, interaction of treatments and ensiling days; SEM, standard error of the mean.

Thus, RL treatment creates an acidic environment with a low pH and many bioactive components. Under anaerobic conditions, this environment facilitates the swift development of helpful microorganisms, particularly LAB, while impeding the development of detrimental microorganisms. In addition, it encourages the rapid accumulation of biogas-producing precursors known as VFAs. However, the effect of adding RP during the anaerobic storage of alfalfa to microbial populations is unknown.

### Bacterial community composition of alfalfa samples during anaerobic storage

**Fig 1:**
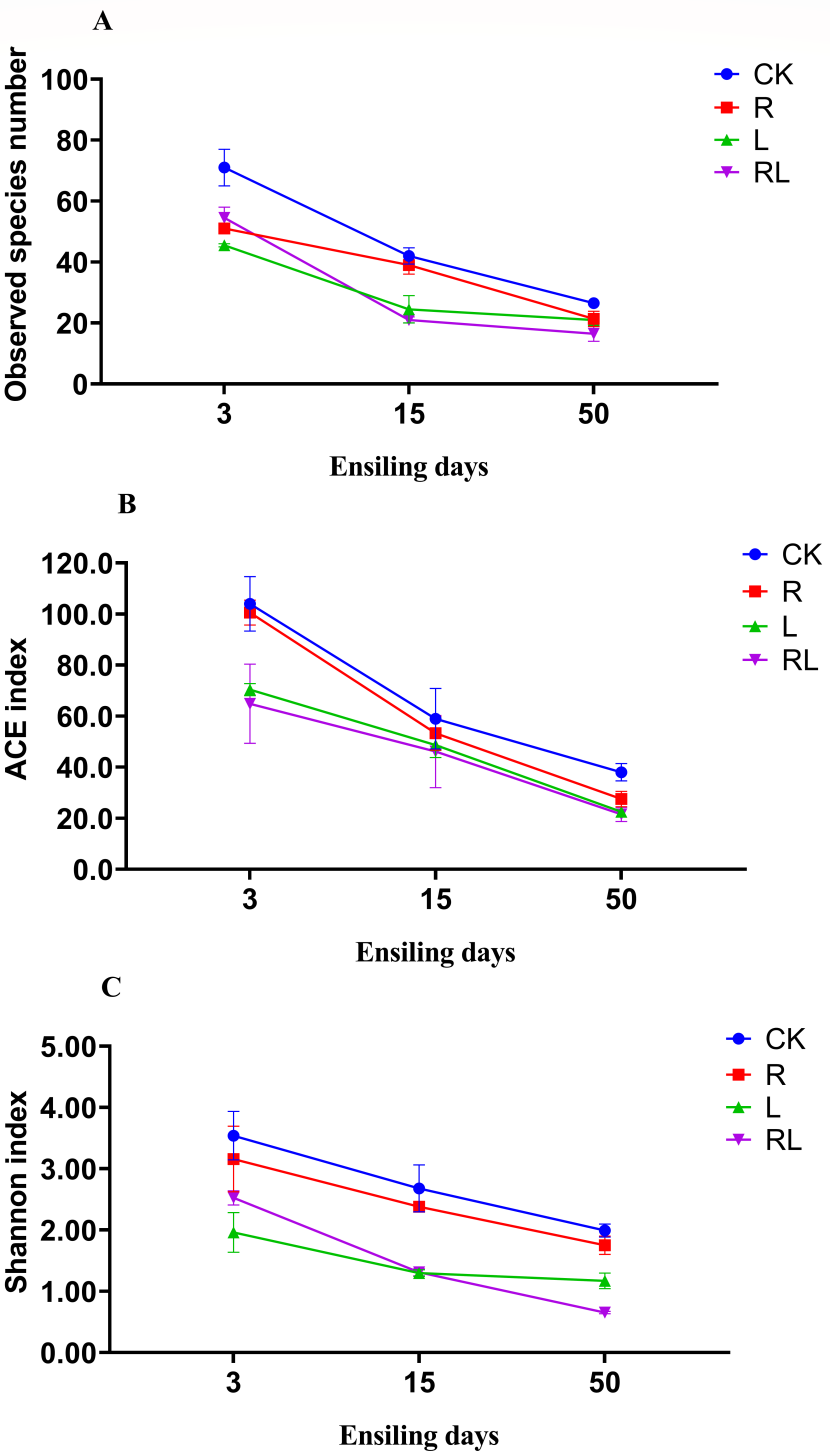
The bacterial alpha-diversity indices (observed species number (**A**), ACE (**B**), and Shannon (**C**)) of alfalfa after 3, 15, and 50 days of anaerobic storage. Without additives (CK); *Rosa roxburghii* pomace (R); *Lactobacillus acidophilus* (L); *Lactobacillus acidophilus* combined with *Rosa roxburghii* pomace (RL); 3, 3 days of anaerobic storage; 15, 15 days of anaerobic storage; and 50, 50 days of anaerobic storage.

Figure 1 displays the alpha diversity of bacteria in the alfalfa samples throughout anaerobic storage. During anaerobic storage, many microorganisms are suppressed because of the acidic environment produced by LAB, and thus, the observed species number decreases sharply (4). Specifically, the lowest number of observed species, observed with the RL treatment, resulted in a decrease in the bacterial diversity indices, including the ACE and Shannon indices, which may be attributed to the bioactive substances of RP as well as the acid produced by *Lactobacillus acidophilus*. The bioactive compounds present in RP, along with the acid generated by *Lactobacillus acidophilus*, exhibited inhibitory effects on a wide range of microorganisms. Consequently, this inhibition resulted in the predominance of LAB, thereby precipitating a reduction in the overall bacterial diversity subsequently. The outcomes of our investigation correspond with the results presented by Chen et al. (24), which indicated that the addition of *Lactiplantibacillus plantarum* and sea buckthorn pomace reduced the bacterial alpha diversity in alfalfa samples. Our findings contradict the outcomes of Zhang et al. (9), who stated that the application of feruloyl esterase-producing *Lactiplantibacillus plantarum* and grape pomace increased bacterial α diversity in alfalfa samples. We speculate that the possible reason for this is that the number of LAB in their samples continues to increase, while the community structure in our samples has stabilized.

**Fig 2:**
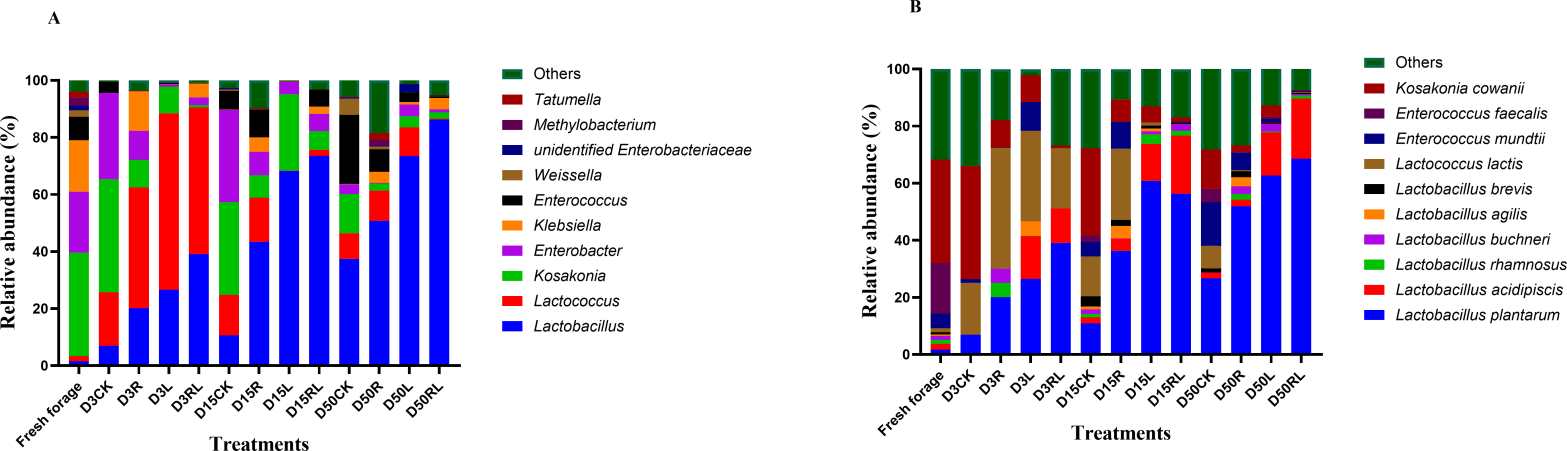
The relative abundance of bacteria at the genus (**A**) and species (**B**) levels in alfalfa after 3, 15, and 50 days of anaerobic storage. Without additives (CK); *Rosa roxburghii* pomace (R); *Lactobacillus acidophilus* (L); *Lactobacillus acidophilus* combined with *Rosa roxburghii* pomace (RL); 3, 3 days of anaerobic storage; 15, 15 days of anaerobic storage; and 50, 50 days of anaerobic storage.

The 10 most prominent bacterial genera in the alfalfa samples during anaerobic storage are shown in Fig. 2A. Overall, *Kosakonia* and *Enterobacter* were found to be dominant in the fresh alfalfa samples. However, after anaerobic storage, the prevalence of *Lactobacillus* and *Lactococcus* increased significantly in the alfalfa samples. Similarly, Chen et al. (24) demonstrated that the genera *Lactobacillus* and *Lactococcus* were the predominant genera in alfalfa samples following anaerobic storage. *Kosakonia* and *Enterobacter* belong to the *Enterobacteriaceae* family, which suggests that anaerobic storage may impede the proliferation of pathogenic bacteria and preserve nutrients. In the alfalfa samples, the relative abundance of *Lactococcus* decreased and that of *Lactobacillus* increased as the anaerobic storage process progressed. Cai et al. (30) carried out a study that provided evidence that LAB with a coccish morphology, primarily consisting of *Lactococcus, Leuconostoc*, *and Weissella*, exhibit robust growth during the first phase of anaerobic fermentation. However, as anaerobic fermentation progresses, these cocci-shaped LAB are gradually replaced by *Lactobacillus* species. An investigation carried out by Chen et al. (24) demonstrated that *Lactobacillus* rapidly became the most predominant genus within legume silage that was inoculated with LAB. During anaerobic storage, treatment with RL resulted in a notable increase in the relative abundance of *Lactobacillus*, accompanied by a decrease in the relative abundance of *Kosakonia* and *Enterobacter* in comparison to that in the CK group. This demonstrated that the RL treatment boosted the proliferation of *Lactobacillus* while inhibiting the proliferation of unwanted bacteria, achieving a relative abundance of *Lactobacillus* greater than 80% of the total bacterial count. The findings of Tang et al. (37) align with our results, which indicated that the relative abundance of LAB in anaerobically fermented perennial sorghum samples was greater than 80% of the total bacterial population.

The top 10 bacterial species in the alfalfa samples during anaerobic storage are displayed in Fig. 2B. In general, the presence of *Kosakonia cowanii* was predominant in samples of freshly harvested alfalfa. Nevertheless, it is essential to emphasize that the relative abundance of *Lactiplantibacillus plantarum* in the alfalfa samples significantly rose following anaerobic storage. The outcome aligns with the findings presented in Fig. 2A. During the process of anaerobic storage, there was a progressive increase in the proportions of *Lactiplantibacillus plantarum* and *Lactobacillus acidophilus* in the alfalfa samples, whereas the relative abundances of *Lactococcus lactis* and *Kosakonia cowanii* decreased. The reason for this is due to the fact that *Lactiplantibacillus plantarum* avoids more decomposition of sugars and proteins in crops by producing LA to rapidly establish an acidic environment, promoting rapid fermentation, and suppressing other harmful bacteria (38). Consistent with the outcomes published by Chen et al. (24) for alfalfa, our results indicate a gradual increase in the proportion of *Lactiplantibacillus plantarum* in alfalfa samples and a decrease in the relative abundance of *Lactococcus lactis*. The primary species identified in the CK treatment were *Lactococcus actis*, *Kosakonia cowanii*, and *Enterococcus mundtii*. The potential reason behind the inferior fermentation quality observed in the CK treatment could be elucidated by this statement. Compared with the CK treatment, the R, L, and RL treatments indicated an elevated relative abundance of *Lactiplantibacillus plantarum* and a decreased relative abundance of *Kosakonia cowanii*. Our outcomes are comparable with those of Chen et al. (32) in their study on alfalfa, where the relative abundance of *Lactiplantibacillus plantarum* was found to be lowest in the CK treatment. This observation suggested that the additives employed in the study had a beneficial influence on the bacterial community composition of the alfalfa samples after anaerobic storage. As expected, at 50 days of anaerobic storage, the RL treatment most significantly influenced the increase in the relative abundance of *Lactiplantibacillus plantarum*, whereas causing a decrease in the relative abundance of *Lactococcus lactis*, *Kosakonia cowanii*, and *Enterococcus mundtii*. Consequently, the bacterial composition of the alfalfa samples after anaerobic storage underwent substantial alterations when subjected to coinoculation with *Lactobacillus acidophilus* and RP, resulting in the creation of an ideal milieu for eventual methane generation. The results of our study align with the conclusions drawn by Tang et al. (37), which stated that pretreatment of perennial sorghum led to alterations in bacterial composition, which consequently increased biogas production. Notably, inoculation with *Lactobacillus acidophilus* resulted in a rise in the relative abundance of *Lactiplantibacillus plantarum*. The current discovery corresponds to the investigation conducted by Weinberg et al. (39), in which they isolated strains of *Lactiplantibacillus plantarum* from sorghum, corn, and alfalfa. Upon coinoculation of these three strains on the crops, it was observed that the strain initially obtained from a particular plant had a greater abundance in the silage derived from that crop than the other two strains. This implies that the optimal LAB for a specific crop can be obtained from that same crop, so we should target the best inoculant to the LAB isolated from that crop. The combination of *Lactobacillus acidophilus* and RP can drive successful anaerobic fermentation by increasing the relative abundance of *Lactiplantibacillus plantarum* in anaerobic fermentation and decreasing the relative abundance of *Kosakonia cowanii*.

**Fig 3:**
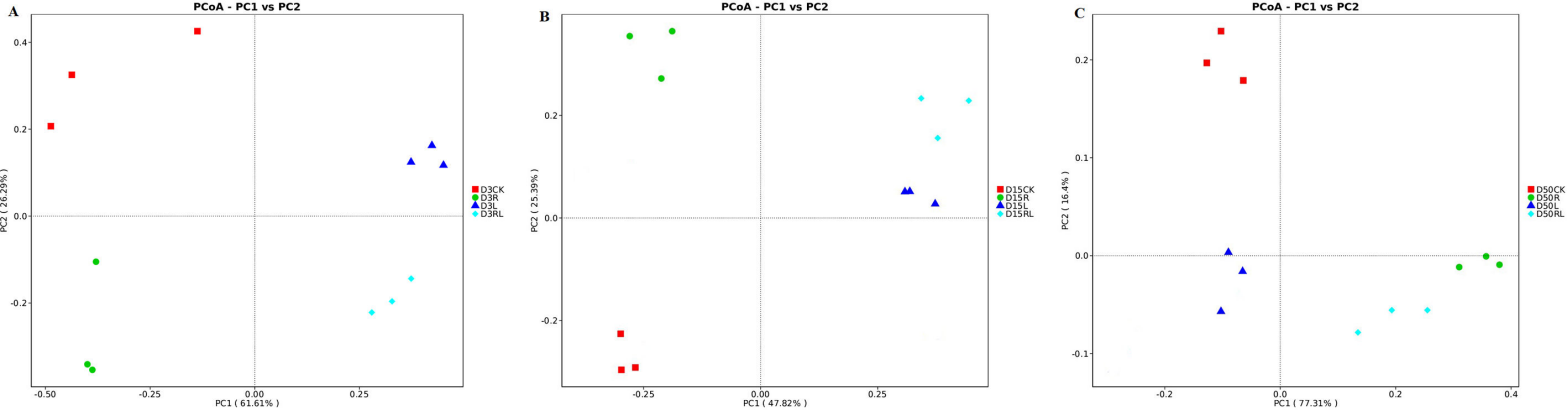
Principal coordinate analysis (PCoA) plot at the bacterial operational taxonomic unit (OTU) level in alfalfa after 3, 15, and 50 days of anaerobic storage (**A–C**). Without additives (CK); *Rosa roxburghii* pomace (R); *Lactobacillus acidophilus* (L); *Lactobacillus acidophilus* combined with *Rosa roxburghii* pomace (RL); 3, 3 days of anaerobic storage; 15, 15 days of anaerobic storage; and 50, 50 days of anaerobic storage.

Figure 3 illustrates the results of PCoA conducted for the levels of bacterial operational taxonomic units (OTUs) during 3, 15, and 50 days of anaerobic storage of alfalfa. According to the PCoA results (Fig. 3A, components 1 and 2 explained 61.61% and 26.29% of the total variance, respectively, after 3 days of anaerobic storage. Based on the PCoA results (Fig. 3B), components 1 and 2 explained 47.82% and 25.39% of the total variance, respectively, after 15 days of anaerobic storage. Concerning the outcomes of the PCoA results (Fig. 3C), components 1 and 2 explained 77.31% and 16.40% of the total variance, respectively, after 50 days of anaerobic storage. During 3 days of anaerobic storage, the bacterial communities among the four alfalfa treatment groups differed greatly. However, during the 15 days of anaerobic storage, the bacterial communities in the L and RL treatments began to resemble each other, and at 50 days of anaerobic storage, the bacterial communities in the R and RL treatments began to resemble each other (Fig. 3C). Conclusions of our investigation align with the outcomes reported by Tang et al. (37), as the control samples were more distant from the other samples, suggesting that there is an influence on the structure of the bacterial community in the inoculant. These results demonstrated that LAB and RP were the major drivers of alterations in the bacterial community of alfalfa samples throughout anaerobic fermentation in the middle and later stages of anaerobic fermentation, respectively.

### Correlations of bacterial communities with ensiling characteristics of alfalfa samples during anaerobic storage

**Fig 4:**
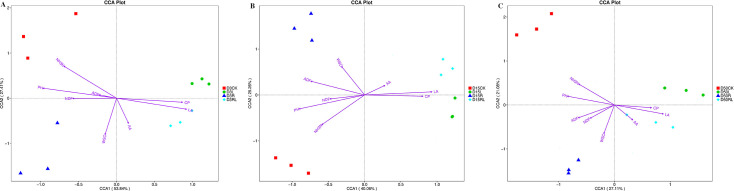
Canonical correlation analysis (CCA) at the bacterial operational taxonomic unit (OTU) level in alfalfa after 3, 15, and 50 days of anaerobic storage (**A-C**). Without additives (CK); *Rosa roxburghii* pomace (**R**); *Lactobacillus acidophilus* (**L**); *Lactobacillus acidophilus* combined with *Rosa roxburghii* pomace (RL); 3, 3 days of anaerobic storage; 15, 15 days of anaerobic storage; and 50, 50 days of anaerobic storage; CP, crude protein; NDF, neutral detergent fiber; ADF, acid detergent fiber; WSC, water-soluble carbohydrate; LA, lactic acid; AA, acetic acid; NH_3_–N, ammonia nitrogen.

Figure 4 shows the results of CCA conducted to examine the associations between the four treatment groups (CK, R, L, and RL treatments) and eight environmental parameters (CP, NDF, ADF, WSC, pH, LA, AA, and NH_3_-N contents). After 3 days of anaerobic storage, there was a substantial positive correlation between the WSC content and the R treatment. In addition, the CP, LA, and AA contents were significantly positively correlated with the RL treatment. Conversely, pH, NH_3_-N, NDF, and ADF contents exhibited significant negative correlations with the RL treatment (Fig. 4A). After 15 days of anaerobic storage, there was a substantial positive correlation between the WSC and ADF contents and the R treatment. In addition, the LA and AA contents significantly positively correlated with the RL treatment. By contrast, pH, NH_3_-N, and NDF content significantly negatively correlated with the RL treatment (Fig. 4B). After 50 days of anaerobic storage, there was a substantial positive correlation between the WSC, NDF, and ADF contents and the R treatment. In addition, the CP, LA, and AA contents were significantly positively correlated with the RL treatment. Conversely, the pH and NH_3_-N content exhibited a significant negative correlation with the RL treatment (Fig. 4C). Moreover, CCA revealed that RP was pivotal in augmenting the WSC content and that the combination of *Lactobacillus acidophilus* and RP may have been essential in enhancing the LA and AA levels as well as preserving the CP and decreasing the NH_3_-N, NDF, and ADF levels. The outcomes obtained in this investigation were similar to the findings presented by Zhang et al. (9).

### Methane yield preservation and anaerobic fermentation characteristics of alfalfa samples

**Fig 5:**
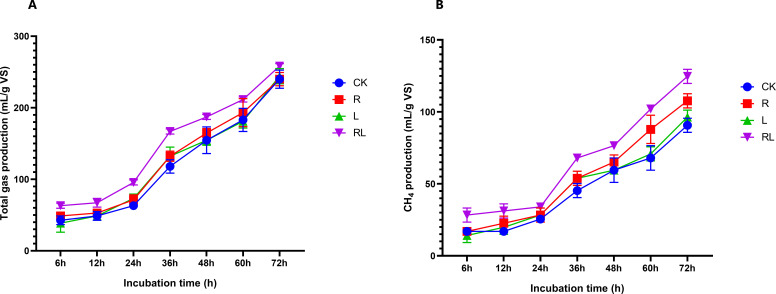
Biogas production (**A**) including methane (**B**) in alfalfa after 50 days of anaerobic storage. Without additives (CK); *Rosa roxburghii* pomace (R); *Lactobacillus acidophilus* (L); *Lactobacillus acidophilus* combined with *Rosa roxburghii* pomace (RL)

*In vitro,* anaerobic fermentation is extensively employed for the assessment of biogas production capacity. Figure 5 displays the gas emissions resulting from the *in vitro* anaerobic fermentation of the alfalfa samples. Since the main component of biogas is methane, we used the production of methane to represent biogas production. The cumulative emissions of gas, including methane, from the alfalfa samples after anaerobic fermentation exhibited a progressive increase. Notably, the most substantial increase in emissions occurred during the time intervals of 24–36 h. This conclusion aligns with the conclusions published by Sun et al. (4), wherein it was shown that the gas production of ear-removed corn exhibited a progressive increase over time and sharply after 24 h of inoculation. The RL treatment, which involved coinoculation with RP and *Lactobacillus acidophilus*, along with the R and L treatments, resulted in higher levels of gas production and biogas yield in comparison to the CK-inoculated samples. The cumulative total gas emissions of the samples inoculated with R were 11.43%, 10.77%, and 0.84% greater than those of the CK-inoculated samples at 24, 36, and 72 h, respectively. The samples inoculated with L exhibited cumulative gas emissions that were 13.89%, 10.77%, and 1.67% greater than the gas emissions of the CK-inoculated samples at 24, 36, and 72 h, respectively. At 24, 36, and 72 h, the cumulative total gas emissions of the RL-inoculated samples were 34.08%, 29.27%, and 14.95% greater than those of the CK-inoculated samples, respectively. Compared with those of the CK-inoculated samples, the cumulative biogas outputs of the R-inoculated samples exhibited significant increases of 10.00%, 15.79%, and 15.79% at 24, 36, and 72 h, respectively. Moreover, compared with those in the CK-inoculated samples, the cumulative biogas production in the L-inoculated samples increased by 10.00%, 15.79%, and 5.88% at 24, 36, and 72 h, respectively. At 24, 36, and 72 h, the cumulative biogas productions of the RL-inoculated samples were 25.00%, 33.33%, and 27.27% greater than that of the CK-inoculated samples, respectively. Our investigation is comparable to that of Tang et al. (37), who documented that the inoculation of *Lactiplantibacillus plantarum* and *Saccharomyces cerevisiae* to perennial sorghum resulted in a significant rise in methane and total gas production. In our investigation, the methane production was decreased compared to Huang et al. (40), who indicated a methane generation of 140 mL/g VS by adding LAB to *Miscanthus sinensis*. The reason for this difference could be that we performed rumen digestion for less time than in their study, which left the methane production incomplete, hence the low production. In our investigation, the methane generation exceeded that observed by Schirmer et al. (41), who reported that fruit and vegetable waste also have some methane production potential. This suggests that mixing waste with lignocellulosic biomass to produce methane increases methane production. The biogas output observed in our investigation for samples inoculated with RL was greater than the biogas production from anaerobically fermented fresh alfalfa grass documented in the research by Amaleviciute et al. (31). The significant conversion of available substrates to VFAs led to increased gas production from rumen fermentation in principle. In addition, the RL treatment resulted in greater total gas and biogas generation, which can be attributed to its elevated WSC content and reduced ADF content (31). Our findings agree with the conclusions of Amaleviciute et al. (31), corroborating that biogas yield is related to the chemical composition of the biomass.

The *in vitro* DM digestibility and VFA content of the alfalfa samples after 72 h of incubation are shown in Table 4. Based on the outcomes of the investigation, the DM digestibility was substantially greater in the R, L, and RL treatments than in the CK treatment. Among these treatments, the RL treatment exhibited the highest level of DM digestibility. Our outcomes contradict the conclusions of Zhang et al. (9), who discovered that alfalfa samples with added grape pomace possessed lower DM digestibility. The reason for this is that the presence of tannins in grape pomace hinders the functioning of digestive enzymes by interacting with proteins, cellulose, hemicellulose, and pectin (42). RP, on the other hand, resulted in higher DM digestibility due to its high dietary fiber content, a prebiotic substance that can undergo fermentation by microorganisms residing in the gastrointestinal system (16). This fermentation process results in the synthesis of short-chain fatty acids, namely, propionate, butyrate, and acetate. This process influences several metabolic pathways, including the absorption of nutrients and carbohydrates and the metabolism of lipids and glucose. Furthermore, it crucially influences the process of colonic anaerobic fermentation, hence influencing the formation of fecal matter. In addition, studies have provided evidence that LAB favorably impacts the digestibility of samples when stored under anaerobic conditions (3). The DM digestibility was greatest in the RL treatment group, which can be attributed to the combined effects of RP and *Lactobacillus acidophilus*. Acetate, propionate, and butyrate are critical VFAs that are produced through carbohydrate fermentation within the rumen. The elevated levels of VFAs generated during *in vitro* incubation can typically be attributed to increased DM degradation in the fermentation substrate. This enhanced degradation provides microorganisms with a greater supply of fermentation substrate, enabling them to create a larger quantity of biogas (27). Our investigation corroborates the outcomes of Tang et al. (37), who also discovered that the VFAs content increased as the *in vitro* culture process progressed. In the current investigation, following a 72 h period of *in vitro* incubation, it was shown that R treatment led to an increase in butyrate concentrations while resulting in a reduction in propionate concentration. After a 72 h period of *in vitro* incubation, the L treatment led to a notable rise in the butyrate concentration while causing a drop in the propionate concentration. In this experiment, after 72 h of *in vitro* incubation, the RL treatment increased the acetate and butyrate concentrations and the acetate/propionate ratio but decreased the propionate concentration. The process of anaerobic storage promotes the build-up of acetate, which, in turn, facilitates subsequent anaerobic fermentation for gas production (4). The substantial presence of biogas in the RL treatment may be ascribed to the efficient conversion of acetate into biogas through anaerobic digestion. Conversely, propionate inhibits the proliferation of methanogenic bacteria, thus the RL treatment results in a greater biogas yield because of the low propionate content (4). While butyrate is typically produced by detrimental microbes during anaerobic fermentation, it can also participate in specific chemical processes that result in biogas generation (14). Our findings indicate that the combination of *Lactobacillus acidophilus* and RP can effectively facilitate anaerobic fermentation by enhancing the levels of acetate and butyrate while suppressing propionate formation. This synergistic effect optimizes the utilization of biomass resources and enhances the generation of biogas.

**TABLE 4:** Dry matter digestibility and VFAs of alfalfa silages at 72 h of incubation *in vitro*^*a*^

Item	CK	R	L	RL	SEM	*P* value
Acetate, mmol/L	26.56^b^	28.95^ab^	26.11^b^	34.57^a^	1.273	0.036
Propionate, mmol/L	12.60^a^	9.12^b^	5.21^c^	4.05^c^	3.597	<0.001
Butyrate, mmol/L	4.14^c^	6.24^b^	5.99^b^	7.93^a^	1.569	0.004
Acetate/propionate	2.11^b^	3.19^b^	5.15^b^	9.28^a^	0.969	0.012
Dry matter digestibility, % DM	37^d^	52^b^	45^c^	59^a^	0.025	<0.001

^*a*^ a−d, means of additive treatments within a column with different superscripts differ in the same ensiling days (*P* < 0.05). DM, dry matter; SEM, standard error of the mean; CK, without additives; R, *Rosa roxburghii* pomace; L, *Lactobacillus acidophilus*; RL, combined *Rosa roxburghii* pomace with *Lactobacillus acidophilus*.

### Conclusion

Overall, coinoculation with *Lactobacillus acidophilus* and RP not only increased the LA and WSC contents compared to the CK treatment but also by boosting the abundance of *Lactobacillus* suppressed the growth of *Kosakonia* and enhanced anaerobic fermentation. The results from the *in vitro* rumen fermentation experiment showed that coinoculation of alfalfa samples with *Lactobacillus acidophilus* and RP increased DM digestibility and intermediate metabolite (mainly acetate) levels, providing more fermentable biomass for biogas production. In addition, the combination of RP and *Lactobacillus acidophilus* application resulted in a beneficial synergistic impact on enhancing biomass retention and biogas production in alfalfa samples. Therefore, coinoculation of RP and *Lactobacillus acidophilus* provides an effective storage and pretreatment strategy for biogas production from alfalfa. The use of ensiling offers an attractive opportunity to increase methane production from fruit waste while reducing costs and synergistically integrating with other pretreatment techniques to optimize the methane generation potential of this waste.
